# Association between Age and Striatal Volume Stratified by CAG Repeat Length in Prodromal Huntington Disease

**DOI:** 10.1371/currents.RRN1235

**Published:** 2011-05-11

**Authors:** Elizabeth Aylward, James Mills, Dawei Liu, Peggy Nopoulos, Christopher A. Ross, Ronald Pierson, Jane S. Paulsen

**Affiliations:** ^*^Center for Integrative Brain Research, Seattle Children's Research Institute; ^†^Department of Psychiatry, The University of Iowa Carver College of Medicine; ^‡^Department of Biostatistics, The University of Iowa, College of Public Health; ^§^Department of Psychiatry, Pediatrics, and Neurology, The University of Iowa Carver College of Medicine and ^¶^Division of Neurobiology, Department of Psychiatry, Johns Hopkins University

## Abstract

Background: Longer CAG repeat length is associated with faster clinical progression in Huntington disease, although the effect of higher repeat length on brain atrophy is not well documented. Method: Striatal volumes were obtained from MRI scans of 720 individuals with prodromal Huntington disease. Striatal volume was plotted against age separately for groups with CAG repeat lengths of 38–39, 40, 41, 42, 43, 44, 45, 46, and 47–54. Results: Slopes representing the association between age and striatal volume were significantly steeper as CAG repeat length increased. Discussion: Although cross-sectional, these data suggest that striatal atrophy, like clinical progression, may occur faster with higher CAG repeat lengths.

## Introduction

It has long been known that greater CAG repeat lengths are associated with earlier onset of illness, especially for individuals with particularly high repeat number. More recent evidence demonstrates that higher CAG repeat lengths are also associated with faster clinical progression. Rosenblatt et al. [1] demonstrated that CAG repeat number is a small but significant predictor of progression rates of HD in four different measures: overall neurologic signs, motor impairment, cognition, and daily function. Evidence for faster progression of striatal atrophy with higher CAG repeat length is not as clear. Cross-sectional and longitudinal studies have yielded conflicting results, with some suggesting that brain atrophy progresses more rapidly for individuals with higher CAG repeat length and others showing no relationship [2] [3] [4] [5] [6] [7] [8]. 

## Materials and Methods

The analyses presented here are based on baseline MRI data from participants of PREDICT-HD, a multi-site, longitudinal study of prodromal HD. The sample included 720 participants who tested positive for the HD gene mutation (CAG repeat lengths ranging from 38 to 54), but had not been diagnosed with the motor signs of HD at the time of study enrollment (“prodromal HD”). An additional 206 participants were offspring of a parent with HD but who themselves had tested negative for the HD gene mutation (“controls”). All aspects of the study were approved by the Institutional Review Board at each participating institution, they were in compliance with the code of Ethics of the World Medical Association Declaration of Helsinki, and all participants gave written informed consent.

            All MRI scans were obtained using a standard multi-modal protocol that included a 3D volumetric spoiled gradient echo series and a dual echo proton density/T2 series. Scans were processed at The University of Iowa using an automated procedure implemented in BRAINS [9] and artificial neural networks [10]. Caudate, putamen, total striatum (caudate + putamen), and total intracranial volumes were obtained.

            Analyses were performed to examine the association between age and striatal volume in each of nine groups defined by CAG repeat length (38–39, 40, 41, 42, 43, 44, 45, 46, 47–54). Each CAG group had at least 34 participants. Table 1 presents demographics and clinical scores for participants in each CAG group. Within each group, a linear regression was performed to examine the association between age and striatal volume (corrected for intracranial volume). The slopes resulting from each of these nine regressions were then correlated with CAG group (using Spearman correlation). This analysis was designed to determine whether the slope of the regressions for age and striatal volume became steeper with increasing CAG repeat length. For each CAG repeat group, a separate linear regression was also performed that included age (centered by group mean to avoid potential multicollinearity issues) and the quadratic term of age as predictors to explore the possibility of a curvilinear relationship between age and striatal volume.

**Table 1.** Sample description and R^2^ of regression between age and striatal volume for each CAG group.

**Table:** 

** **	**Control**	** Prodromal HD Participants Grouped by CAG Repeat Length**								
CAG length		38–39	40	41	42	43	44	45	46	47–54
N	206	55	108	123	135	108	60	62	35	34
Mean age (s.d.)	43.8 (11.7)	49.6 (11.5)	47.5 (9.5)	44.9 (9.5)	40.8 (7.9)	39.0 (7.1)	36.4 (7.0)	35.4 (6.5)	33.7 (7.9)	30.4 (3.9)
Age range	19.1–83.7	20.8–77.8	23.6–67.9	26.0–67.7	24.9–58.1	20.3–56.8	20.1–51.6	22.2–50.0	27.4–50.1	23.4–39.3
Gender (% female)	64.1	61.8	65.7	65.8	59.3	64.8	58.3	54.8	74.3	76.5
Mean UHDRS Motor Score (s.d.)	NA	3.6 (4.7)	4.3 (4.4)	5.2 (5.5)	4.4 (4.8)	5.1 (5.4)	5.7 (8.0)	6.4 (7.8)	7.9 (7.4)	9.4 (7.5)
R-Square *p* for regression	0.15 (<0.0001)	0.14 (0.004)	0.28 (<0.0001)	0.41 (<0.0001)	0.48 (<0.0001)	0.43 (<0.0001)	0.51 (<0.0001)	0.39 (<0.0001)	0.36 (<0.0001)	0.27 (0.002)

## Results

            Figure 1 shows the regression for each group depicting the association between age and striatal volume (corrected for intracranial volume). These regressions were all highly significant (R^2^s ranging from 0.14 to 0.51, all *p* values < 0.005) but variable, with lower R^2^s generally observed for the lowest CAG repeat lengths. The slope for each group (representing association between striatal volume and age) was highly associated with CAG group (Spearman *r* = −0.98, *p* < 0.0001; see Figure 2), with higher CAG repeat numbers associated with steeper slope, at least up through CAG = 44. 

            The quadratic effect of age in the linear regression model was statistically significant for CAG = 46 group (*t* = 2.85, *p* = 0.008), although this was due to a single outlier. When this outlier was removed, the addition of the age^2^ factor did not result in an increased significance in the model that was based on age alone (*t* = 0.21, *p* = 0.83 for age^2^, after accounting for age). Although not quite reaching significance (*t* = −1.85, *p* = 0.07), the curve for the CAG = 38–39 group suggested a slightly steeper decline for older subjects than younger subjects. A significant effect of age^2^ was not observed in any other groups (*p* values all > 0.20). 

**Figure:**
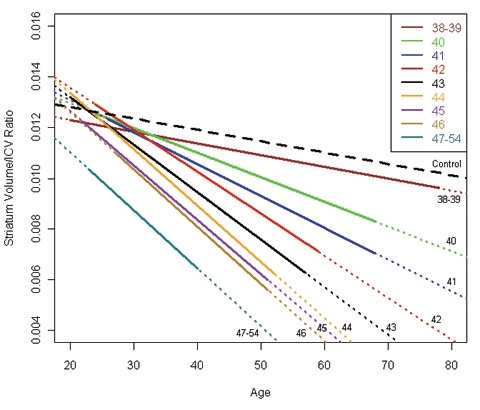
**Figure 1.** Regressions for age and striatal volume (corrected for ICV) for each CAG group. The solid portion of each line represents actual data (based on age range of participants in each CAG group). Heavy dashed line represents correlation for control participants.

**Figure:**
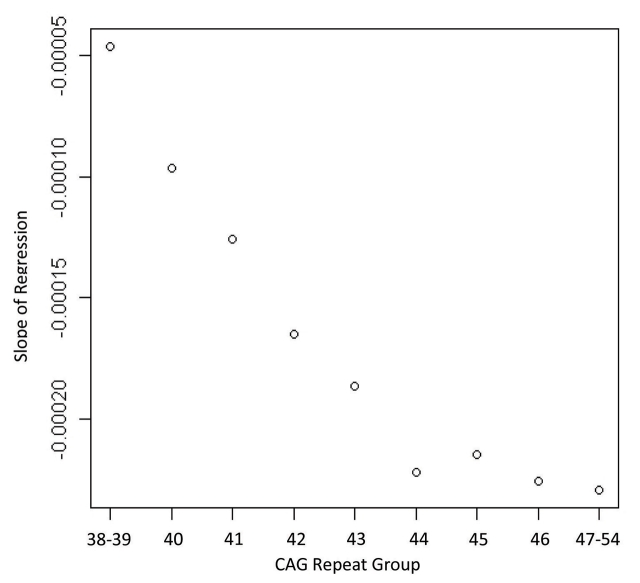
**Figure 2. **Correlation showing the relationship between the slopes of the regression lines from Figure 1 and the CAG repeat lengths of each group (Spearman correlation = 0.98, *p* < 0.0001)

** **

## Discussion

Although based on cross-sectional data, our analyses suggest that increased CAG repeat length is associated with faster progression of striatal atrophy in prodromal HD, at least up through CAG = 44. Although it is clearly established that CAG repeat length has an effect on age at onset of HD [1] [11],few studies have examined the effect of CAG repeat length on rate of brain atrophy, and these have all been done on relatively small samples and most have examined brain regions other than the striatum. In a longitudinal study of 37 affected patients, Ruocco et al. [7] found that higher repeat length (> 45) was associated with faster rate of atrophy in frontal, occipital, parietal, and cerebellar regions. In a small sample (*n* = 13) including both affected and prodromal individuals, Henley et al. [5] found no significant association between rate of whole brain atrophy and CAG repeat length. In a larger sample (*n* = 62), the same group found that an increase of CAG repeat length by one was associated with an increase in whole-brain atrophy rate of 0.12% per year [6]. In small samples that included both prodromal and affected subjects, Squitieri et al. [8] found a significant correlation between CAG repeat length and increased CSF volume change, and Aylward et al. [2] found that repeat length correlated significantly with rate of change in caudate, globus pallidus, and total basal ganglia, but not putamen. One cross-sectional MRI study with a small sample also demonstrated a significant correlation between CAG repeat length and striatal volume loss (difference between HD subject’s volume and control volume [4]), while a neuropathological study of established HD found a correlation between CAG and cortical, but not subcortical atrophy [3].

Our results are consistent with analyses of longitudinal data from a subsample of the current cross-sectional sample (*n* = 211 [12]) that revealed a significant association between CAG repeat length and rate of change for caudate (*t* = −2.64, *p *= 0.009) and total striatum (*t* = −2.32, *p* = 0.02), with a trend toward a significant association for putamen (*t* = −1.80, *p *= 0.07). No significant associations were observed for any other regions (cortical gray matter, white matter, CSF, thalamus). Taken together with results from the current study, these findings yield evidence suggesting that rate of striatal atrophy is faster in individuals with higher CAG repeat lengths. Our results are not surprising, given previous research in affected patients with HD demonstrating that (a) faster rate of clinical progression is associated with higher CAG repeat number [1] and (b) smaller striatal volumes are associated with more severe clinical manifestations [13].

            A major strength of the current study is its large sample size. Although the findings presented here are based on cross-sectional baseline data, it is expected that longitudinal results would be similar, as the regression between age and striatal volume for a given CAG repeat length can be assumed to be a good estimate of the trajectory of atrophy for the average participant within that CAG group. Lack of very young participants (< 18 years) may skew the data somewhat, especially for the longer CAG groups, where the *y*-intercept is below that of the other CAG groups (see Figure 1). There is also a lack of cases with very high CAG repeat lengths, as these individuals usually have childhood onset and would not, therefore, qualify for a study of adult prodromal HD. If it were possible to include prodromal individuals younger than 18 years, striatal volumes for those with large CAG repeat lengths might be higher than those in the current study, resulting in even steeper slopes for these groups. Thus, our finding of similar association between age and striatal volume in the groups with CAG > 44 may not be valid across the entire age range. 

            It is also noteworthy that the slope for the group with CAG = 38–39 is basically the same as for the control group, although absolute values for striatal volumes are lower. The age range for the two groups is similar and the results were not biased by any obvious outliers. The trend for a curvilinear relationship between age and striatal volume (*p* = 0.07) suggests that striatal atrophy remains fairly normal for prodromal individuals with relatively low CAG repeat lengths until they are older adults, at which time atrophy increases. This would be consistent with the fact that these individuals are usually not diagnosed until fairly late in life.

Evidence that individuals with longer CAG repeat lengths show faster striatal atrophy may be important in the design of future clinical trials in prodromal HD. By selecting participants with relatively longer CAG repeat lengths and faster rate of atrophy, clinical trials might be able to be conducted with smaller sample sizes or shorter duration than selecting those with relatively shorter CAG repeat lengths and slower rate of atrophy.

## Acknowledgments

We thank the PREDICT-HD sites, the study participants, and the National Research Roster for Huntington Disease Patients and Families. The complete list of those involved in the PREDICT-HD study is below.

## Funding Information

This research is supported by the National Institutes for Health, National Institute of Neurological Disorders and Stroke (NS40068) and CHDI Foundation, Inc.

## Competing Interests

The authors have declared that no competing interests exist.

**PREDICT-HD Investigators, Coordinators, Motor Raters, Cognitive Raters **(as of January 5, 2010)

Peg Nopoulos, MD, Robert Rodnitzky, MD, Ergun Uc, MD, BA, Leigh J. Beglinger, PhD, Vincent A. Magnotta, PhD, Stephen Cross, BA, Nicholas Doucette, BA, Andrew Juhl, BS, Jessica Schumacher, BA, Mycah Kimble, BA, Pat Ryan, MS, MA, Jessica Wood, MD, PhD, Eric A. Epping, MD, PhD, Thomas Wassink, MD, and Teri Thomsen, MD (University of Iowa Hospitals and Clinics, Iowa City, Iowa, USA); David Ames, MD, Edmond Chiu, MD, Phyllis Chua, MD, Olga Yastrubetskaya, PhD, Joy Preston, Anita Goh, D.Psych, and Angela Komiti, BS, MA (The University of Melbourne, Kew, Victoria, Australia);  Lynn Raymond, MD, PhD, Rachelle Dar Santos, BSc, Joji Decolongon, MSC, and David Weir, BSc (University of British Columbia, Vancouver, British Columbia, Canada);  Adam Rosenblatt, MD, Christopher A. Ross, MD, PhD, Barnett Shpritz, BS, MA, OD, and Claire Welsh (Johns Hopkins University, Baltimore, Maryland, USA);  William M. Mallonee, MD and Greg Suter, BA (Hereditary Neurological Disease Centre, Wichita, Kansas, USA);  Ali Samii, MD, Hillary Lipe, ARNP, and Kurt Weaver, PhD (University of Washington and VA Puget Sound Health Care System, Seattle, Washington, USA);  Randi Jones, PhD, Cathy Wood-Siverio, MS, Stewart A. Factor, DO, and Claudia Testa, MD, PhD (Emory University School of Medicine, Atlanta, Georgia, USA);  Roger A. Barker, BA, MBBS, MRCP, Sarah Mason, BSC, Anna Goodman, PhD, and Anna DiPietro (Cambridge Centre for Brain Repair, Cambridge, UK); Elizabeth McCusker, MD, Jane Griffith, RN, and Kylie Richardson, PhD (Westmead Hospital, Sydney, Australia);  Bernhard G. Landwehrmeyer, MD, Daniel Ecker, MD, Patrick Weydt, MD, Michael Orth MD, PhD, Sigurd Süβmuth, MD, RN, Katrin Barth, RN, and Sonja Trautmann, RN (University of Ulm, Ulm, Germany); Kimberly Quaid, PhD, Melissa Wesson, MS, and Joanne Wojcieszek, MD (Indiana University School of Medicine, Indianapolis, IN); Mark Guttman, MD, Alanna Sheinberg, BA, Adam Singer, and Janice Stober, BA, BSW (Centre for Addiction and Mental Health, University of Toronto, Markham, Ontario, Canada);  Susan Perlman, MD and Arik Johnson, PsyD (University of California, Los Angeles Medical Center, Los Angeles, California, USA);  Michael D. Geschwind, MD, PhD and Jon Gooblar, BA (University of California San Francisco, California, USA); Tom Warner, MD, PhD, Stefan Klöppel, MD, Maggie Burrows, RN, BA, Marianne Novak, MD, Thomasin Andrews, MD, BSC, MRCP, Elisabeth Rosser, MBBS, FRCP, and Sarah Tabrizi, BSC, PhD (National Hospital for Neurology and Neurosurgery, London, UK);  Anne Rosser, MD, PhD, MRCP and Kathy Price, RN (Cardiff University, Cardiff, Wales, UK);  Amy Chesire, LCSW-R, MSG, Frederick Marshall, MD, and Mary Wodarski, BA (University of Rochester, Rochester, New York, USA);  Oksana Suchowersky, MD, FRCPC, Sarah Furtado, MD, PhD, FRCPC, and Mary Lou Klimek, RN, BN, MA (University of Calgary, Calgary, Alberta, Canada);  Peter Panegyres, MB, BS, PhD, Carmela Connor, BP, MP, DP, and Elizabeth Vuletich, BSC (Neurosciences Unit, Graylands, Selby-Lemnos & Special Care Health Services, Perth, Australia);  Joel Perlmutter, MD and Stacey Barton, MSW, LCSW (Washington University, St. Louis, Missouri, USA);  Sheila A. Simpson, MD, Daniela Rae, RN, and Zosia Miedzybrodzka, PhD (Clinical Genetics Centre, Aberdeen, Scotland, UK);  David Craufurd, MD, Ruth Fullam, BSC, and Elizabeth Howard, MD (University of Manchester, Manchester, UK)  Pietro Mazzoni, MD, PhD, Karen Marder, MD, MPH, Carol Moskowitz, MS, and Paula Wasserman, MA (Columbia University Medical Center, New York, New York, USA);  Diane Erickson, RN, Dawn Miracle, BS, MS, and Rajeev Kumar, MD (Colorado Neurological Institute, Englewood, Colorado, USA);  Vicki Wheelock, MD, Terry Tempkin, RNC, MSN, Nicole Mans, BA, MS, and Kathleen Baynes, PhD (University of California Davis, Sacramento, California, USA);  Joseph Jankovic, MD, Christine Hunter, RN, CCRC, and William Ondo, MD (Baylor College of Medicine, Houston, Texas, USA);  Justo Garcia de Yebenes, MD, Monica Bascunana Garde, Marta Fatas, BA, and Jose Luis Lópenz Sendon, MD (Hospital Ramón y Cajal, Madrid, Spain);  Martha Nance, MD, Dawn Radtke, RN, and David Tupper, PhD (Hennepin County Medical Center, Minneapolis, Minnesota, USA);  Wayne Martin, MD, Pamela King, BScN, RN, and Satwinder Sran, BSC (University of Alberta, Edmonton, Alberta, Canada);  Anwar Ahmed, PhD, Stephen Rao, PhD, Christine Reece, BS, Janice Zimbelman, PhD, PT, Alexandra Bea, BA, and Emily Newman, BA (Cleveland Clinic Foundation, Cleveland, Ohio, USA); **Steering Committee **Jane Paulsen, PhD, Principal Investigator, Eric A. Epping, MD, PhD, Douglas Langbehn, MD, PhD, Hans Johnson, PhD, Megan Smith, PhD, Janet Williams, PhD, RN, FAAN (University of Iowa Hospitals and Clinics, Iowa City, IA); Elizabeth Aylward, PhD (Seattle Children's Research Institute, WA); Kevin Biglan, MD (University of Rochester, Rochester, NY); Blair Leavitt, MD (University of British Columbia, Vancouver, BC, Canada); Marcy MacDonald, PhD (Massachusetts General Hospital); Martha Nance, MD (Hennepin County Medical Center, Minneapolis, MN); Jean Paul Vonsattel, PhD (Columbia University Medical Center, New York, NY).  **Scientific Sections Bio Markers:** Blair Leavitt, MDCM, FRCPC (Chair) and Michael Hayden, PhD (University of British Columbia); Stefano DiDonato, MD (Neurological Insitute “C. Besta,” Italy); Ken Evans, PhD (Ontario Cancer Biomarker Network); Wayne Matson, PhD (VA Medical Center, Bedford, MA); Asa Peterson, MD, PhD (Lund University, Sweden), Sarah Tabrizi, PhD (National Hospital for Neurology and Neurology and Neurosurgery, London).  **Cognitive:** Deborah Harrington, PhD (Chair, University of California, San Diego), Tamara Hershey, PhD (Washington University Cognitive Science Battery Development); Holly Westervelt, PhD (Chair, Quality Control and Training, Alpert Medical School of Brown University), Jennifer Davis, PhD, Pete Snyder, PhD, and Geoff Tremont, PhD, MS (Scientific Consultants, Alpert Medical School of Brown University); Megan Smith, PhD (Chair, Administration), David J. Moser, PhD, Leigh J. Beglinger, PhD (University of Iowa); Lucette Cysique, PhD (St. Vincent’s/University of Melbourne, Australia); Carissa Gehl, PhD (VA Medical Center, Iowa City, IA); Robert K. Heaton, PhD, David Moore, PhD, Joanne Hamilton, PhD, and David Salmon, PhD (University of California, San Diego); Kirsty Matheson (University of Aberdeen); Paula Shear, PhD (University of Cincinnati); Karen Siedlecki, PhD (Fordham University); Glenn Smith, PhD (Mayo Clinic); and Marleen Van Walsem (EHDN). **Functional Assessment:** Janet Williams, PhD (Co-Chair), Leigh J. Beglinger, PhD, Anne Leserman, MSW, LISW, Justin O’Rourke, MA, Bradley Brossman, MA, Eunyoe Ro, MA (University of Iowa); Rebecca Ready, PhD (University of Massachusetts); Anthony Vaccarino, PhD (Ontario Cancer Biomarker Network); Sarah Farias, PhD (University of California, Davis); Noelle Carlozzi, PhD (Kessler Medical Rehabilitation Research & Education Center); and Carissa Gehl, PhD (VA Medical Center, Iowa City, IA). **Genetics:** Marcy MacDonald, PhD (Co-Chair), Jim Gusella, PhD, and Rick Myers, PhD (Massachusetts General Hospital); Michael Hayden, PhD (University of British Columbia); Tom Wassink, MD (Co-Chair) and Eric A. Epping, MD, PhD (University of Iowa). **Imaging:** *Administrative:* Ron Pierson, PhD (Chair), Kathy Jones, BS, Jacquie Marietta, BS, William McDowell, AA, Steve Dunn, BA, Greg Harris, BS, Eun Young Kim, MS, and Yong Qiang Zhao, PhD (University of Iowa); John Ashburner, PhD (Functional Imaging Lab, London); Vince Calhoun, PhD (University of New Mexico); Steve Potkin, MD (University of California, Irvine); Klaas Stephan, MD, PhD (University College of London); and Arthur Toga, PhD (University of California, Los Angeles). *Striatal:* Elizabeth Aylward, PhD (Chair, Seattle Children's Research Institute) and Kurt Weaver, PhD (University of Washington and VA Puget Sound Health Care System, Seattle, Washington). *Surface Analysis:* Peg Nopoulos, MD (Chair), Eric Axelson, BSE, and Jeremy Bockholt, BS (University of Iowa). *Shape Analysis:* Christopher A. Ross (Chair), MD, PhD, Michael Miller, PhD, and Sarah Reading, MD (Johns Hopkins University); Mirza Faisal Beg, PhD (Simon Fraser University). *DTI:* Vincent A. Magnotta, PhD (Chair, University of Iowa); Karl Helmer, PhD (Massachusetts General Hospital); Kelvin Lim, MD (University of Ulm, Germany); Mark Lowe, PhD (Cleveland Clinic); Sasumu Mori, PhD (Johns Hopkins University); Allen Song, PhD (Duke University); and Jessica Turner, PhD (University of California, Irvine).  *fMRI:* Steve Rao, PhD (Chair), Erik Beall, PhD, Katherine Koenig, PhD, Mark Lowe, PhD, Michael Phillips, MD, Christine Reece, BS, and Jan Zimbelman, PhD, PT (Cleveland Clinic). **Motor:** Kevin Biglan, MD (University of Rochester), Karen Marder, MD (Columbia University), and Jody Corey-Bloom, MD, PhD (University of California, San Diego) all Co-Chairs; Michael Geschwind, MD, PhD (University of California, San Francisco); and Ralf Reilmann, MD (Muenster, Germany). **Psychiatric:** Eric A. Epping, MD, PhD (Chair), Nancy Downing, RN, MSN, Jess Fiedorowicz, MD, Robert Robinson, MD, and Megan Smith, PhD (University of Iowa); Karen Anderson, MD (University of Maryland); David Craufurd, MD (University of Manchester); Mark Groves, MD (Columbia University); Anthony Vaccarino, PhD and Ken Evans, PhD (Ontario Cancer Biomarker Network); Hugh Rickards, MD (Queen Elizabeth Psychiatric Hospital); and Eric van Duijn, MD (Leiden University Medical Center, Netherlands). **Core Sections Statistics:** Douglas Langbehn, MD, PhD (Chair) and James Mills, MEd, MS (University of Iowa); and David Oakes, PhD (University of Rochester). **Recruitment/Retention:** Martha Nance, MD (Chair, University of Minnesota); Anne Leserman, MSW, LISW, Stacie Vik, BA, Christine Anderson, BA, Nick Doucette, BA, Kelly Herwig, BA, MS, Mycah Kimble, BA, Pat Ryan, MSW, LISW, MA, Jessica Schumacher, BA, Kelli Thumma, BA, and Elijah Waterman, BA (University of Iowa); and Norm Reynolds, MD (University of Wisconsin, Milwaukee). **Ethics:** Cheryl Erwin, JD, PhD, (Chair, McGovern Center for Health, Humanities and the Human Spirit); Eric A. Epping, MD, PhD and Janet Williams, PhD (University of Iowa); and Martha Nance, MD (University of Minnesota). **IT/Management:** Hans Johnson, PhD (Chair), R.J. Connell, BS, Paul Allen, AASC, Sudharshan Reddy Bommu, MS, Karen Pease, BS, Ben Rogers, BA, BSCS, Jim Smith, AS, Kent Williams, BSA, MCS, MS, Shuhua Wu, MCS, and Roland Zschiegner (University of Iowa). **Program Management Administrative:** Chris Werling-Witkoske (Chair), Karla Anderson, BS, Kristine Bjork, BA, Ann Dudler, Jamy Schumacher, Sean Thompson, BA (University of Iowa). **Financial:** Steve Blanchard, MSHA (Co-Chair), Machelle Henneberry, and Kelsey Montross, BA (University of Iowa).
